# Analysis of Vibration Signals Based on Machine Learning for Crack Detection in a Low-Power Wind Turbine

**DOI:** 10.3390/e25081188

**Published:** 2023-08-09

**Authors:** Angel H. Rangel-Rodriguez, David Granados-Lieberman, Juan P. Amezquita-Sanchez, Maximiliano Bueno-Lopez, Martin Valtierra-Rodriguez

**Affiliations:** 1 ENAP-Research Group, CA-Sistemas Dinámicos y Control, Facultad de Ingeniería, Universidad Autónoma de Querétaro (UAQ), Campus San Juan del Río, Río Moctezuma 249, Col. San Cayetano, San Juan del Río 76807, Mexico; 2 ENAP-Research Group, CA-Fuentes Alternas y Calidad de la Energía Eléctrica, Departamento de Ingeniería Electromecánica, Tecnológico Nacional de México/ITS de Irapuato, Carretera Irapuato-Silao km 12.5, Colonia El Copal, Irapuato 36821, Mexico; 3 Departamento de Electrónica, Instrumentación y Control, Universidad del Cauca, Popayán 190002, Colombia

**Keywords:** ANOVA, blades, cracks, k-nearest neighbors, machine learning, statistical features, vibrations, wind turbines

## Abstract

Currently, renewable energies, including wind energy, have been experiencing significant growth. Wind energy is transformed into electric energy through the use of wind turbines (WTs), which are located outdoors, making them susceptible to harsh weather conditions. These conditions can cause different types of damage to WTs, degrading their lifetime and efficiency, and, consequently, raising their operating costs. Therefore, condition monitoring and the detection of early damages are crucial. One of the failures that can occur in WTs is the occurrence of cracks in their blades. These cracks can lead to the further deterioration of the blade if they are not detected in time, resulting in increased repair costs. To effectively schedule maintenance, it is necessary not only to detect the presence of a crack, but also to assess its level of severity. This work studies the vibration signals caused by cracks in a WT blade, for which four conditions (healthy, light, intermediate, and severe cracks) are analyzed under three wind velocities. In general, as the proposed method is based on machine learning, the vibration signal analysis consists of three stages. Firstly, for feature extraction, statistical and harmonic indices are obtained; then, the one-way analysis of variance (ANOVA) is used for the feature selection stage; and, finally, the k-nearest neighbors algorithm is used for automatic classification. Neural networks, decision trees, and support vector machines are also used for comparison purposes. Promising results are obtained with an accuracy higher than 99.5%.

## 1. Introduction

The generation of energy through renewable sources has significantly increased in recent decades [1], especially currently, as caring for the environment is an important issue [2]. In particular, wind energy is exploited via wind turbines (WTs) [3,4]. Among the main components of a WT, the blades are of utmost importance as they account for approximately 22.2% of the total cost [5]. In this regard, the study of blade damage is crucial for timely maintenance and reducing replacement costs during operation. The early detection of blade damage can also extend the useful life of WTs, reduce the costs of maintenance, and minimize downtime [6,7]. Different factors can impact the structural integrity of WT blades, including corrosive environments, temperature changes, and mechanical stresses from wind profiles and harsh weather conditions. These factors can produce small cracks that, if left unattended, can propagate and lead to catastrophic blade failures [8,9,10,11]. Therefore, the development and application of methods to automatically detect cracks in the blades of WTs and characterize their severity are of paramount importance.

The analysis of vibration signals is a common approach to assess the condition of blades since it allows us to examine their dynamic response to external forces, such as wind profiles [12,13,14,15]. It is worth noting that this dynamic response changes if a blade presents some modifications in its structure; therefore, vibration signals can provide valuable information for damage detection [16]. In this regard, several studies have focused on detecting vibration changes in order to perform WT condition monitoring. For example, Ha et al. [17] presented a methodology that computes the rotor speed and the power of a WT for the classification of different operating conditions; then, their obtained results were correlated with the ones obtained from a vibration signal analysis. Colone et al. [18] proposed a vibration-based method that detects changes in the mass of blades using their natural frequencies and two statistical tests. Wang et al. [19] performed a vibration analysis based on the ensemble empirical mode decomposition (EEMD) and fast Fourier transform (FFT) methods to determine the natural frequencies of the blades of a WT and, thus, assess its dynamic status. All these authors demonstrate and conclude that vibration-based methods are a powerful tool for condition monitoring.

Within the field of WT condition monitoring studies, researchers have also employed machine learning (ML) techniques [20], using methods such as neural networks, support vector machines, and decision trees [21], which are computationally efficient methods to be implemented either offline or online, even using low-end processors. Other advantages such as their low design complexity and ease of implementation have been highlighted in other fields such as structural health monitoring [22] and condition monitoring in machining [23]. In addition, optimization strategies are also integrated to further improve computational costs, as is the case of the work of Tang et al. [24], which uses the kNN algorithm along with the optimum combination of three parameters for the intelligent diagnosis of wind turbine blades. The KNN along with eigenvalue perturbation techniques (EPTs) and features such as recursive Mahalanobis distance and recursive residual error have shown excellent results for the real-time detection of WT downtimes [25]. In the above work, five classes (i.e., in operation, with faults, without wind, under maintenance, and other events) from two wind farms located in Ireland were considered. In particular, EPTs have received special attention because they have been proven to be robust and efficient compared to other methods, exceling in dealing with data uncertainty, assisting in the identification of system parameters, and enabling real-time operation [26,27]. Regarding real-time monitoring systems, the recursive singular spectrum analysis (RSSA) method has been also reported in the literature for structural damage detection from vibration signals. In addition, the RSSA’s advantages, such as its replicability, scalability, and transferability, have allowed for its integration into a single-sensor-based fault detection solution [27,28,29]. As for ML techniques, they are based on obtaining relevant indicators from physical variables and selecting an appropriate classification algorithm for automatic diagnoses. Wang et al. [21] proposed a ML framework for the detection of cracks through images taken by unmanned aerial vehicles (UAVs), in which the Haar-like features and a cascading classifier are used; within this classifier, the LogitBoost method, decision trees, and support vector machines are used. Joshuva et al. [22] carried out a study of the condition of the blades of a WT by using statistical indicators, a J48 decision tree algorithm for feature selection, and the best-first tree algorithm and functional trees as classifiers. Shihavuddin et al. [23] presented an automatic analysis of images with the help of ML to detect damage in WT blades by using convolutional neural networks. Xu et al. [30] proposed clustering by fast search and find of density peaks (CFSFDP) for the identification of different damage modes by using acoustic emissions. Joshuva et al. [31] conducted a study to discriminate different faults in WT blades by using histogram features, a J48 decision tree algorithm, and different lazy classifiers, i.e., the nearest neighbor, k-nearest neighbors, locally weighted learning, and the K-star classifier, of which the best results were obtained by the locally weighted learning. From these previously reviewed works, it is evident that promising results have been obtained, demonstrating the potential of ML techniques; however, one crucial aspect that has not been thoroughly investigated is the impact of wind turbine (WT) velocity on the performance of ML strategies for blade damage detection. To further advance our understanding of this topic, it is crucial to explore how WT velocity affects the effectiveness and accuracy of classifiers. Specifically, there is a need to investigate how the features extracted from vibration signals change under different WT velocities and different damage severities, as well as how these variations impact the performance of the classifiers, looking for a strategy that can be applied regardless of the WT velocity.

In order to contribute to the solution of the previously mentioned problem and harness the full potential of ML capabilities and the information provided by vibration signals, this work investigates and evaluates the effectiveness of a ML scheme for the detection of cracks in a WT blade by using vibration signals, in which three different levels of severity and three different levels of wind velocity are considered. Firstly, for feature extraction, statistical indicators, impulse metrics, and signal processing metrics are computed over the monitored vibration signals. Secondly, the obtained features are ranked from most to least relevant by using a one-way analysis of variance (ANOVA). Lastly, the KNN method is used as a pattern recognition method for the ranked indicators. It is worth noting that, in this last stage, a balance between the obtained accuracy by the KNN method and its computational load (i.e., the number of selected features) is sought. To compare the KNN performance, decision trees (DTs), neural networks (NNs), and support vector machines (SVMs) are also tested. These methods have been used in both the works mentioned in the previous paragraph and in fault diagnoses of rotating machines [24]. The obtained results demonstrate that the proposed ML strategy is a promising tool for crack detection with an accuracy higher than 99.5%.

## 2. Theoretical Background

This section briefly introduces the concepts and algorithms used in the presented work.

### 2.1. Wind Turbine

**Figure 1:**
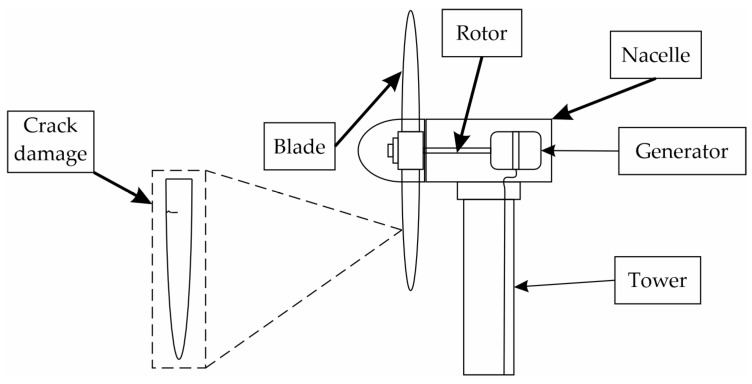
Horizontal WT.

A WT is an electrical machine that converts wind energy into kinetic energy and subsequently converts kinetic energy into electrical energy. WTs are usually classified into two main types: vertical and horizontal WTs, with the latter being one of the most common. This classification is based on the orientation of their axis. Generally, a WT comprises several blades, a rotor, a generator, and a nacelle [7,32]. Figure 1 shows a horizontal five-blade WT, which also shows an example of a crack damage.

As shown in Figure 1, the crack damage changes the blade structure, altering its vibrational response.

#### Vibration Model

Vibration signals are widely used for monitoring the health of a WT. They reflect its dynamics. Every time the blade passes through the tower of the WT, it causes a vibration, called the tower effect; if some abnormalities that generate an imbalance appear (e.g., cracks), a new set of vibrations will appear. The obtained vibration model is as follows [33]:

(1) $$v\left(t\right)=\sum_{m=1}^{M}A_{m}\sin\left(m\omega t+\varphi_{m}\right)+\sum_{n=1}^{N}A_{n}\sin\left(B\omega t+\varphi_{n}\right)$$

where the first section is caused by the rotor imbalance and the second section is caused by the tower effect. $A_{m}$ and $A_{n}$ are the *m* and *n* amplitudes of the vibrations caused in the rotor by the imbalance and by the tower effect, respectively; B is the number of blades; ω is the rotational speed of the rotor; and $\varphi_{m}$ and $\varphi_{n}$ are the *m*th and *n*th initial phases of each vibration component.

From this model, it is possible to assume that the changes in the vibrations can be characterized through a set of different indicators/features in order to distinguish different WT conditions.

### 2.2. Signal Features

Signal features can be used for the detection of changes that may indicate the status of the signal or the status of a system. These changes can be characterized by using statistical features, impulsive metrics, and signal processing metrics [20]. During the analysis of these indicators, it is expected that the obtained data will vary according to the WT conditions.

#### 2.2.1. Statistical Features

Statistical features mainly rely on formulas that allow us to obtain the characteristics of the signal. Among these features, the mean, the standard deviation, and the root mean square (RMS) value are found. Additionally, there are indicators that provide information about the distribution and shape of the signal, such as kurtosis and skewness. 

The following formulas are used, where *X* represents a finite-length vector and N is the vector size [34]:

**Mean**: the average value of a segment or vector given by the following formula:

(2) $$\bar{x}=\frac{1}{N}\sum_{i=1}^{N}X_{i}$$

**RMS**: the value of the amplitude that is related to the amount of energy of the signal. It is calculated by the following formula:

(3) $$X_{rms}=\sqrt{\frac{1}{N}\sum_{i=1}^{N}{\left|X_{i}\right|}^{2}}\;$$

**Standard deviation**: the positive square root of the variance in relation to the mean of the data according to the following equation:

(4) $$S=\sqrt{\frac{\sum_{i=1}^{N}{\left(X_{i}-\bar{x}\right)}^{2}}{N-1}}\;$$

**Shape factor**: the signal shape independent of the signal dimensions.

(5) $$X_{sf}=\frac{X_{rms}}{\frac{1}{N}\sum_{i=1}^{N}\left|X_{i}\right|}$$

**Kurtosis**: the degree of the concentration of the values of a variable around the central zone of the signal distribution.

(6) $$X_{kurt}=\frac{\frac{1}{N}\sum_{i=1}^{N}{\left(X_{i}-\bar{x}\right)}^{4}}{{\left(\frac{1}{N}\sum_{i=1}^{N}{\left(X_{i}-\bar{x}\right)}^{2}\right)}^{2}}$$

**Skewness**: the asymmetry or symmetry of the data according to their distribution.

(7) Skw=1N∑i=1Nxi−x¯31N∑i=1Nxi−x¯232 

#### 2.2.2. Impulsive Metrics

Impulsive metrics characterize the peaks of the signal.

**Peak value:** the maximum absolute value of the signal.

(8) $$X_{p}=max|X_{i}|$$

**Crest factor:** the relation between the peak value and the RMS levels of a signal.

(9) $$C=\frac{X_{p}}{\sqrt{\frac{1}{N}\sum_{i=1}^{N}x_{i}^{2}}}$$

**Impulse factor:** the height of a peak value with the mean value of the signal.

(10) $$X_{if}=\frac{X_{p}}{\frac{1}{N}\sum_{i=1}^{N}\left|X_{i}\right|}$$

**Clearance factor:** the height of a peak value with the squared mean value of the square roots of the absolute values of the signal samples.

(11) $$L=\frac{X_{p}}{{\left[\frac{1}{N}\sum_{i=1}^{N}\sqrt{\left|X_{i}\right|}\right]}^{2}}\;$$

#### 2.2.3. Signal Processing Metrics

The signal processing metrics are functions that characterize the distortion of a signal. The deterioration of the system can cause an increase in noise and a change in the harmonic content. 

**Signal-to-noise ratio (SNR)**: the ratio between the desired information or the power of a signal and the undesired signal or the power of the background noise.

(12) $$SNR=\frac{P_{signal}}{P_{noise}}$$

**Total harmonic distortion (THD)**: the ratio between the harmonic content and the fundamental component of the analyzed signal.

(13) $$THD=\frac{\sqrt{\sum_{i=2}^{n}X_{i}^{2}}}{X_{1}}$$

**Signal-to-noise and distortion ratio (SINAD)**: a measure of quality related the SNR and THD.

(14) $$SINAD=10\;*\;log\frac{1}{10^{\frac{-SNR}{10}}+10^{\frac{THD}{10}}}$$

As can be expected, all the previously mentioned signal features can have different performance values according to the nature of the signal or system, including different operating conditions, e.g., a damage condition. In this regard, it is necessary to sort or rank the features in order to determine which feature or features provide more information about the signal or system.

### 2.3. One-Way Analysis of Variance (ANOVA)

A one-way ANOVA allows us to discover whether different groups of an independent variable impact the response variable y in a different way [35]. This method is a linear model defined as follows:

(15) $$y_{ij}=\alpha_{j}+\varepsilon_{ij}\;$$

where $y_{ij}$ are independent observations, in which i represents the observation number and j represents a different group, $\alpha_{j}$ represents the mean for the $j^{th}$ group, and $\varepsilon_{ij}$ is the random error.

In general, an ANOVA helps us determine if the constants, $\alpha_{j}$, are all the same. Therefore, it tests the hypothesis that the means of all the groups are the same, as opposed to the alternative hypothesis that at least one group differs from the others. This criterion can be used to observe which feature or features provide more discriminant information to differentiate among classes/operating conditions. After the features have been ranked according to their relevance by using an ANOVA (i.e., a feature selection method), they can be selected and used for pattern recognition through ML classifiers.

### 2.4. Machine Learning Classifiers

In supervised ML, labeled datasets are used to train algorithms that classify new outcomes. In this work, DTs, SVMs, and KNN- and NN-based algorithms are explored.

#### 2.4.1. Decision Tree

**Figure 2:**
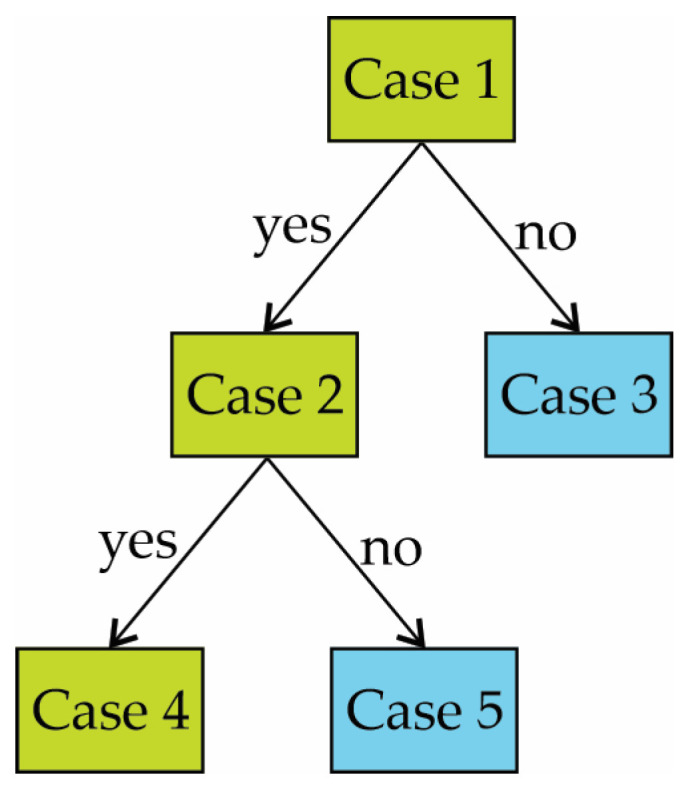
Simple decision tree model.

A decision tree is a prediction model that consists of inductively learning from observations or conditions and logical constructions. It has a great similarity with rule-based predictions used for categorizing different types of data. The learning process is represented by a tree graph that contains a set of nodes and branches. The main node is the attribute from which the process starts, and the internal nodes correspond to each of the cases to be solved [36]. Figure 2 shows a basic model of decision trees, wherein a particular path is chosen based on whether the required conditions are met. The statistical procedures to construct DTs can be found in [36].

#### 2.4.2. Support Vector Machine

**Figure 3:**
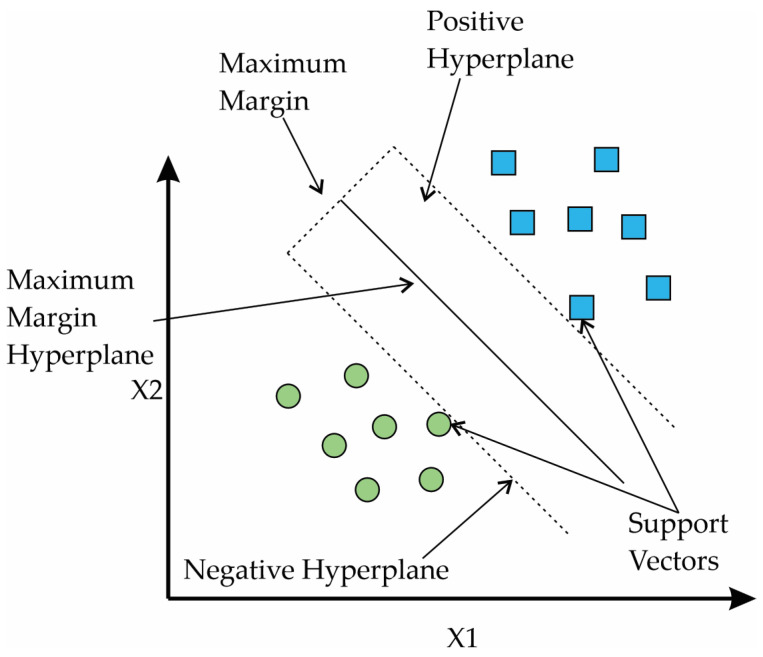
Sections of a Support Vector Machine.

SVMs are a set of supervised learning algorithms that have emerged as classification and regression methods. They operate on a dataset of dimension *n* and map it to a higher-dimensional space using a kernel function. This transformation allows the data to be treated as a linear problem in the new space, effectively solving the problem without considering the original data dimensionality [37]. In this regard, it is a linear classifier that seeks an optimal hyperplane between two distinct classes to derive a decision function for classifying samples into specific classes. The decision function is constructed by using the following formula [38]:

(16) $$g\left(x\right)=\omega^{T}\;x+\omega_{0}=0\;$$

where ω is the vector of the weights, x is the input vector, and $\omega_{0}$ is the bias. Figure 3 shows the sections of an SVM.

#### 2.4.3. K-Nearest Neighbors

**Figure 4:**
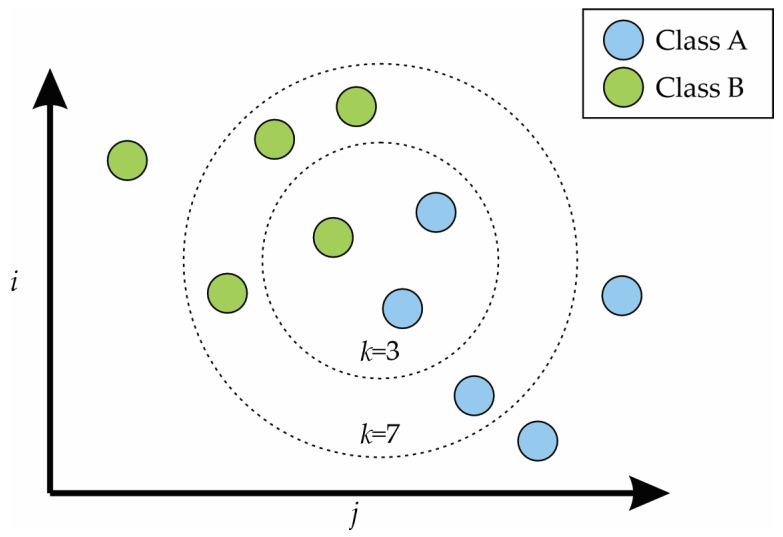
K-nearest neighbors with *k* = 3 and *k* = 7.

Among the ML classifiers, the KNN algorithm is a method used for classification according to space characteristics. This algorithm is considered one of the simplest algorithms in the field of ML because the assignment is carried out by a majority vote of the nearest neighbors, and the object (or set of inputs) is assigned to the most common class among its k-nearest neighbors [39]. The mathematical model used is as follows [40]:

(17) $$\hat{y_{i}}=\sum_{j=1}^{k}b_{j}y_{P\left(i,j\right)}\;$$

where $\hat{y_{i}}$ represents the output estimation, $P\left(i,j\right)$ is the index of the *j*th nearest neighbor for the sample $y_{i}$, and b represents the results of the Moore–Penrose inverse. Figure 4 shows an example of the k-nearest neighbors with *k* = 3 and *k* = 7. 

#### 2.4.4. Neural Network

**Figure 5:**
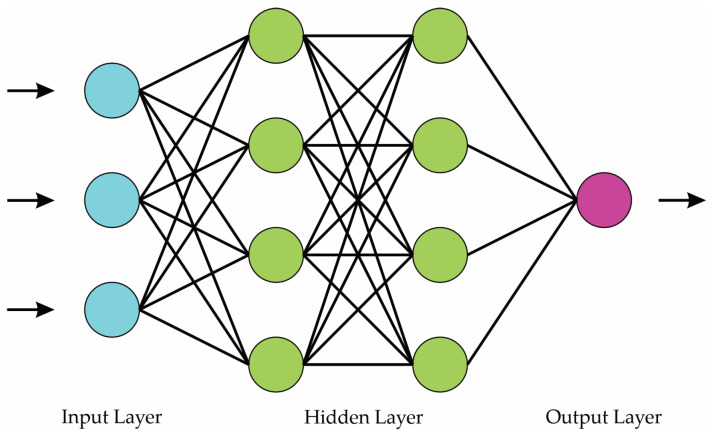
Neural network model.

NNs are systems capable of learning to solve problems by recognizing patterns. They emulate the structure of the human brain, which enables them to perform pattern recognition tasks [41]. In this structure, information flows from the input layer to the output nodes, passing through the hidden layer. To characterize the network weights, input/output data pairs are presented. A training rule is then applied to adjust these weights. The training process aims to minimize the error between the desired and calculated outputs and continues iteratively until the overall error is deemed acceptable. The mathematical model that describes each neuron is as follows:

(18) $$y=f\left(\sum_{i=1}^{I}w_{i}x_{i}+b\right)$$

where y is the output, w represents the weights, *x* represents the inputs, b is the bias, f(·) is the activation function, and I is the total number of inputs. Figure 5 shows a typical configuration of an NN, which is composed of an input layer, a hidden layer, and an output layer.

## 3. Methodology

**Figure 6:**
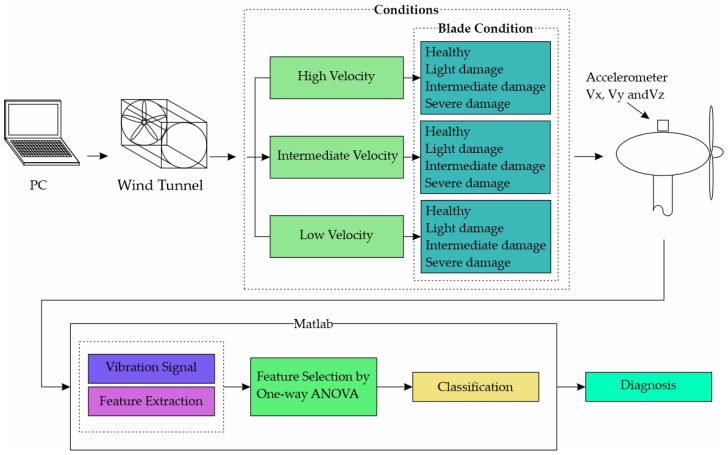
Proposed methodology.

The proposed methodology is shown in Figure 6. In general, the methodology consists of 3 stages. The first stage involves the obtaining of the vibration signals for the different blade conditions (i.e., healthy, light damage, intermediate damage, and severe damage) and different wind velocities (i.e., low, intermediate, and high velocity) measured by WT rps (revolutions per second). In all these cases, the signals are acquired in steady state and for the three axes (Vx, Vy, and Vz) through an accelerometer located at the top of the WT nacelle. In the second stage, the signals are processed to obtain the indicators mentioned in Section 2.2 for each axis. These indicators are separated by velocity, considering 4 conditions (healthy, light damage, intermediate damage, and severe damage). These indicators are then ranked/sorted using ANOVA, prioritizing the most relevant ones for classifying the different damage conditions. Finally, once the indicators have been obtained and sorted/ranked, the most significant ones are used in different ML classifiers to achieve the best results, using the smallest possible number of indicators. The entire process is repeated for each velocity using Matlab software. Three velocities, i.e., low, intermediate, and high, are selected for the operating range of the WT.

## 4. Experimental Setup and Results

### 4.1. Experimental Setup

**Figure 7:**
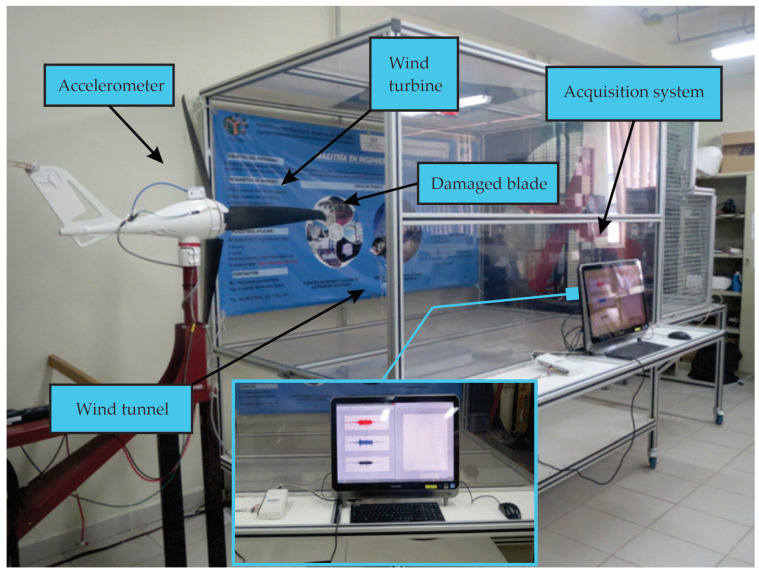
Experimental setup.

Figure 7 shows the experimental setup used in the development of this work. It includes a wind tunnel, which is used to generate the wind profiles (i.e., the three different velocities: 4 rps, 8 rps, and 12 rps in a steady state). The low-power WT is an air X model with 12 V and 400 W. For the crack damages, a healthy blade is gradually damaged. To mitigate disturbances caused by the wind tunnel, the WT is mounted on an external base, securely fixed to the ground.

In order to acquire the vibration signals of the WT, the accelerometer is mounted on the WT nacelle. The accelerometer used is a KISTLER model 8395A10. For data acquisition, a National Instruments (NI) USB-6211 board at a sampling rate of 10,000 samples/s is used. The computer used for conducting these tests has the following hardware specifications: a CPU with 2.30 GHz, 16 GB RAM, and a 64-bit operating system. The implementation software for the overall methodology is MATLAB 2022a.

### 4.2. Crack Information

**Figure 8:**
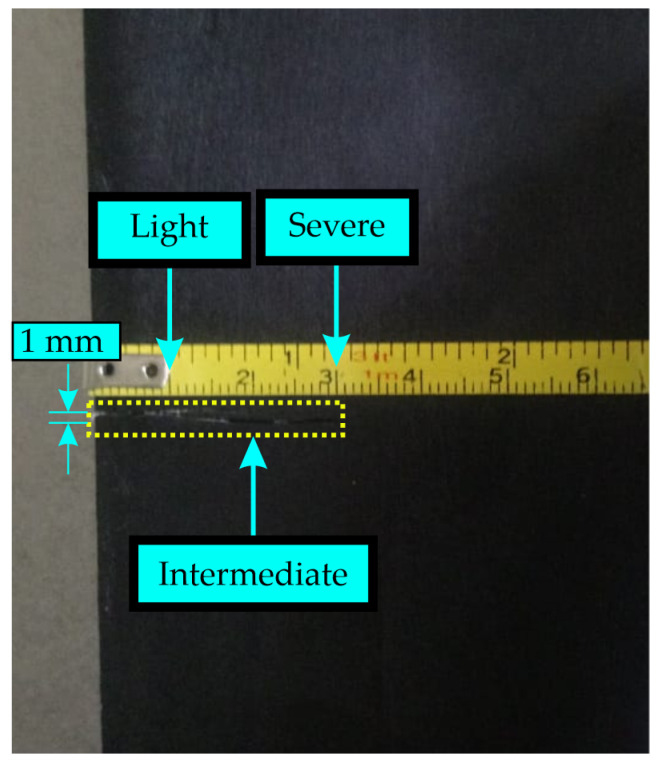
Crack on the blade.

As mentioned previously in the methodology section, this study involves working with blades that have a notch at one end. This notch simulates a crack and its progression across four conditions: healthy, light, intermediate, and severe (0 cm, 1 cm, 2 cm, and 3 cm, respectively, with a cutting width of 1 mm). The total width of the blade is 7 cm. The notch is made with a fretsaw. For each severity level, the depth of the notch is increased to simulate the advancement of the crack. As can be seen in Figure 8, the cut is barely visible, making it difficult to perceive with the naked eye. However, it is of vital importance to detecting the presence of the crack and determining its severity accurately.

### 4.3. Vibration Signals

Table 1 describes the dataset used in this work. There are four blade conditions: healthy, light damage, intermediate damage, and severe damage. For each blade condition, there are three velocities: low, intermediate, and high, in which 100 vibration signals are acquired for the three axes, i.e., X, Y, and Z, giving a total of 1200 signals for each axis. The three velocities correspond to 4 rps (i.e., 240 rpm or a low velocity), 8 rps (i.e., 480 rpm or an intermediate velocity), and 12 rps (i.e., 720 rpm or a high velocity). With these values, the entire range of the WT velocity (i.e., from ~3 to 12 rps) is taken into account. For all these cases, the WT starts at 0 rps but the tests continue until it reaches the in-test speeds and maintains a steady state.

**Figure 9:**
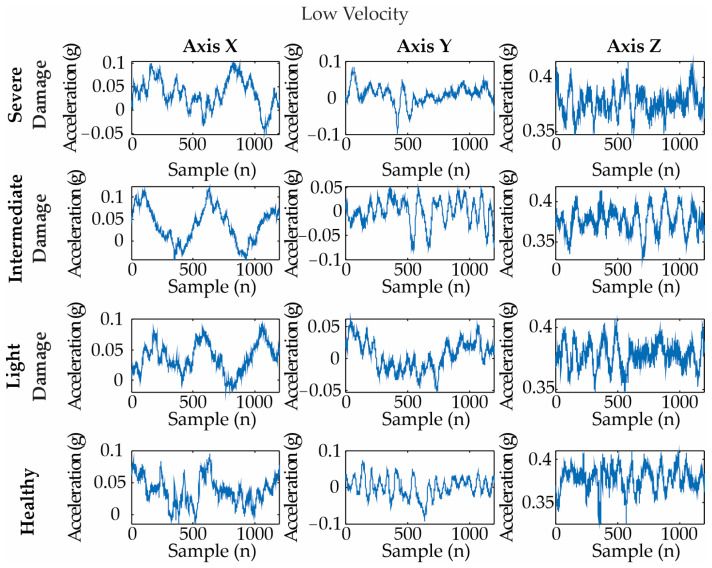
Vibration signals for different blade conditions under a low velocity (i.e., 4 rps). They are measured as an acceleration in gravity: g = m/s^2^.

**Figure 10:**
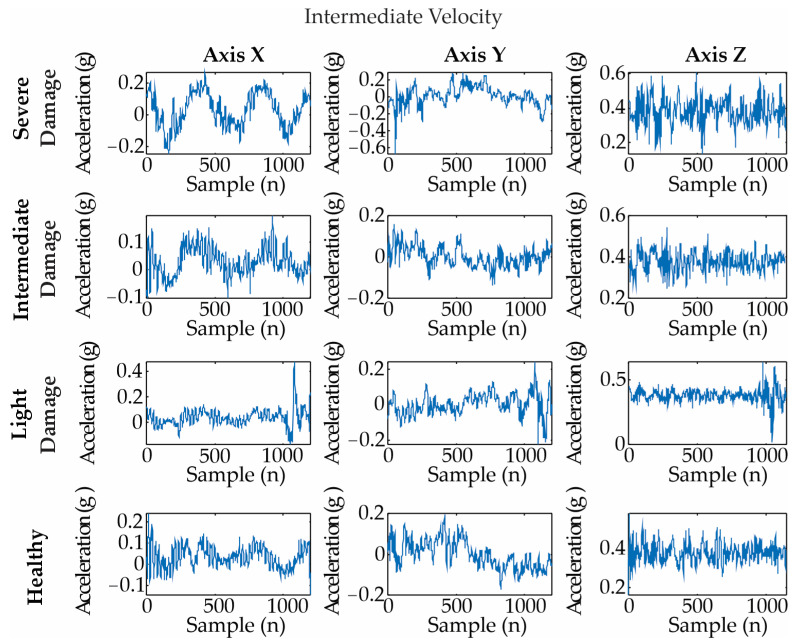
Vibration signals for different blade conditions under an intermediate velocity (i.e., 8 rps). They are measured as an acceleration in gravity: g = m/s^2^.

**Figure 11:**
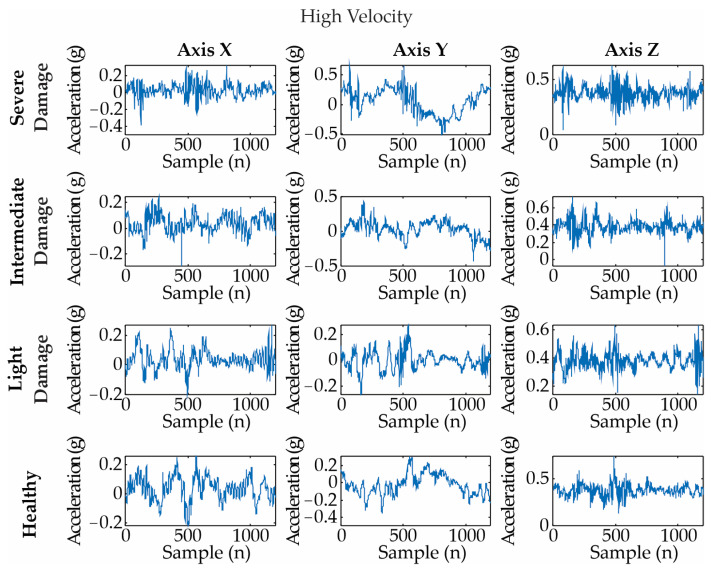
Vibration signals for different blade conditions under a high velocity (i.e., 12 rps). They are measured as an acceleration in gravity: g = m/s^2^.

Figure 9, Figure 10 and Figure 11 show examples of the acquired vibration signals for 4 rps, 8 rps, and 12 rps, respectively. Each analyzed signal has 1200 length samples, i.e., 0.12 s.

### 4.4. Statistical Feature Selection

**Figure 12:**
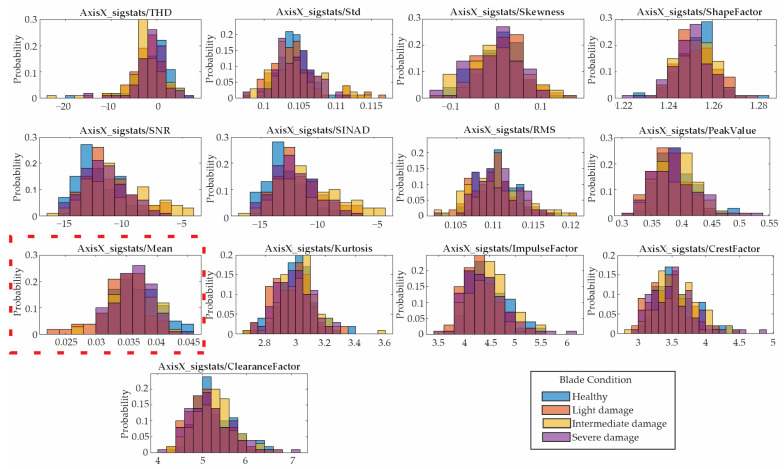
Histograms of the indicators obtained from the X-axis.

**Figure 13:**
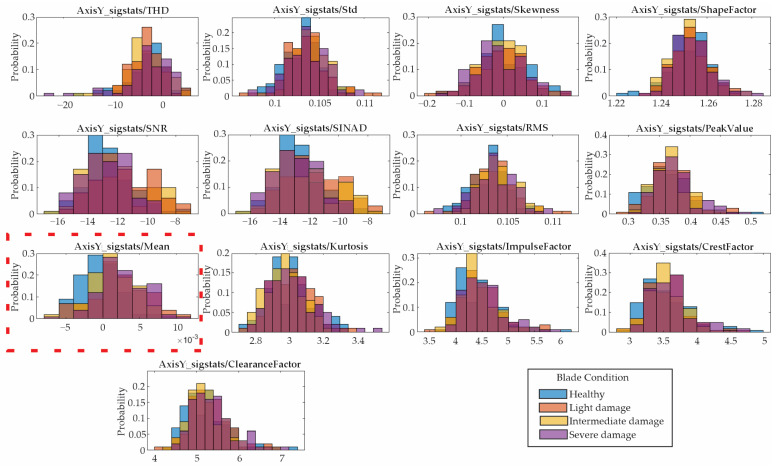
Histograms of the indicators obtained from the Y-axis.

**Figure 14:**
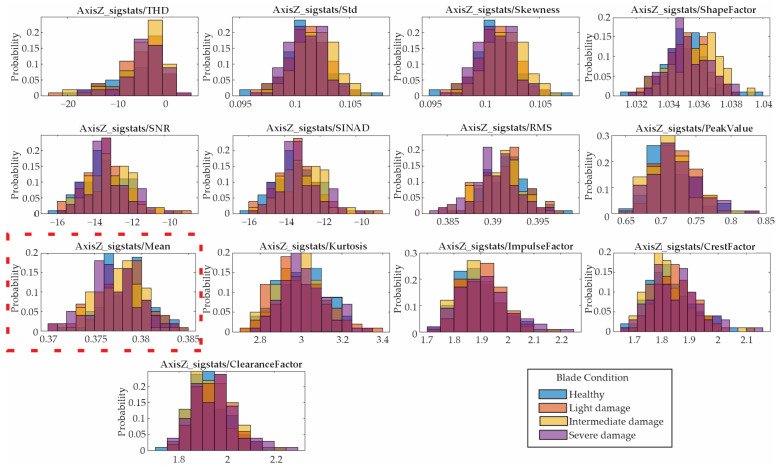
Histograms of the indicators obtained from the Z-axis.

Following the proposed methodology, the 13 features presented in Section 2.2 are computed for the vibration signals described in the previous section. Due to the amount of data, only the results for the low velocity are shown. Figure 12, Figure 13 and Figure 14 show the obtained results in form of histograms for the X-axis, Y-axis, and Z-axis, respectively. The histograms show the frequency distribution of the data; in this regard, the non-overlapped zones in Figure 12, Figure 13 and Figure 14 allow us to distinguish between the blade conditions. For instance, the histograms for the mean values (marked with dotted red rectangles) show that there are some non-overlapped zones that can contribute to the differentiation between the different blade conditions, e.g., the orange color for the X-axis/Figure 12 for light damage, the blue color for the Y-axis/Figure 13 for healthy, and the yellow color for the Z-axis/Figure 13 for intermediate damage. Some colors seem darker due to their overlap. Despite the non-overlapped zones, there are many regions that present some overlaps, avoiding linear separation and consequently requiring the application of pattern recognition algorithms.

**Figure 15:**
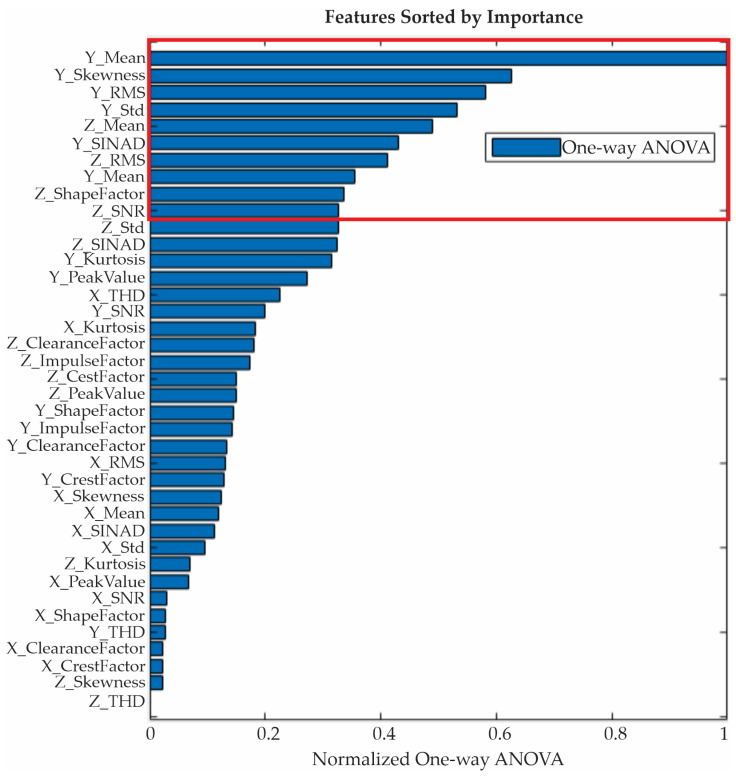
One-way ANOVA results.

In order to quantitatively determine the contribution of each feature for distinguishing the blade conditions, an ANOVA test is applied. Figure 15 shows the obtained results. In this graph, the indicators are ranked and ordered in terms of their relevance for identifying each severity condition, with the mean for the Y-axis being the most discriminant. For the next stage in the proposed method, i.e., the classification stage, only the first ten features, marked by the red rectangle, are used. Table 2 shows their numerical values. It is worth noting that the use of 10 features is decided in a heuristic way; however, other strategies to select the most appropriate number of features can be also implemented. 

### 4.5. Classifiers

**Figure 16:**
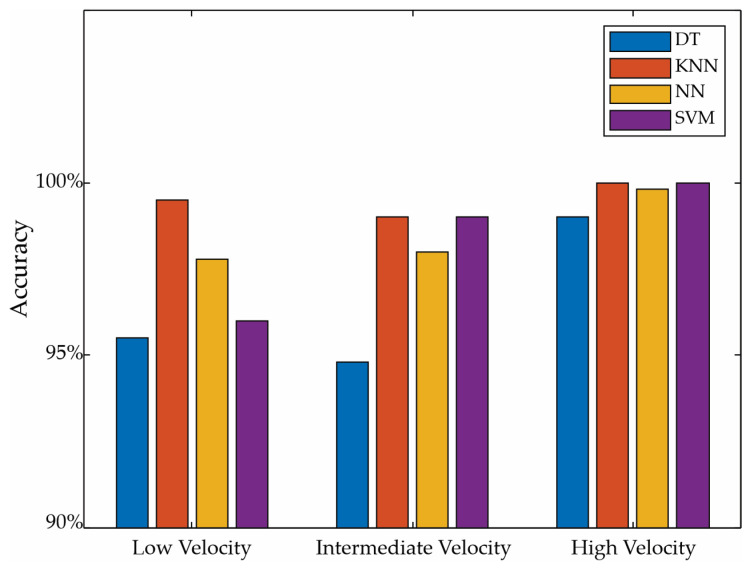
Classification’s accuracy.

After ordering the indicators, a comparison of different ML classifiers, such as decision trees, k-nearest neighbors, vector support machines, and neural networks, was carried out. In this regard, the four classifiers are applied to the indicators of each velocity in a separated way. To achieve a balance between the accuracy and computational load, all of the 10 indicators were initially used and then gradually reduced by removing the less relevant indicators. After making this reduction, the best results were obtained using three indicators (i.e., the mean, RMS, and standard deviation) for each velocity. It is worth noting that they correspond to the same axis, reducing the number of channels of the sensor for future implementation. The obtained accuracy in each velocity can be seen in Figure 16. For instance, at low velocities, a 95.5% accuracy for DTs is obtained; a 96% accuracy for SVMs with the Gaussian kernel function, with a kernel scale of 0.43 and a one-vs-one multiclass method, is obtained; a 97.8% accuracy for a feedforward NN with ReLU as the activation function and one hidden layer of 100 neurons is obtained; a 99.5% accuracy for the KNN is obtained with *k* = 5, an Euclidian distance metric, and an equal distance weight. At high velocities, the four classifiers achieve accuracies higher than 99.5%. In all of the classifiers, a cross validation of five was used, which is a typical value for small datasets. It is worth noting that the hyper-parameters used in the classifiers were selected by testing different standard configurations, such as linear, quadratic, cubic, and Gaussian kernels for the SVMs, Euclidean, cosine, and Minkowski (cubic) distance metrics for the KNN method, and ReLU and the sigmoid activation function for the NNs. The best results were obtained with the KNN method using *k* = 5, an Euclidian distance metric, and an equal distance weight. Although suitable results were obtained, some optimization strategies to determine the best settings for the classifiers were used. Although, in general, similar and good results are obtained in all of the classifiers, the best results for the three velocities are obtained by using the KNN.

**Figure 17:**
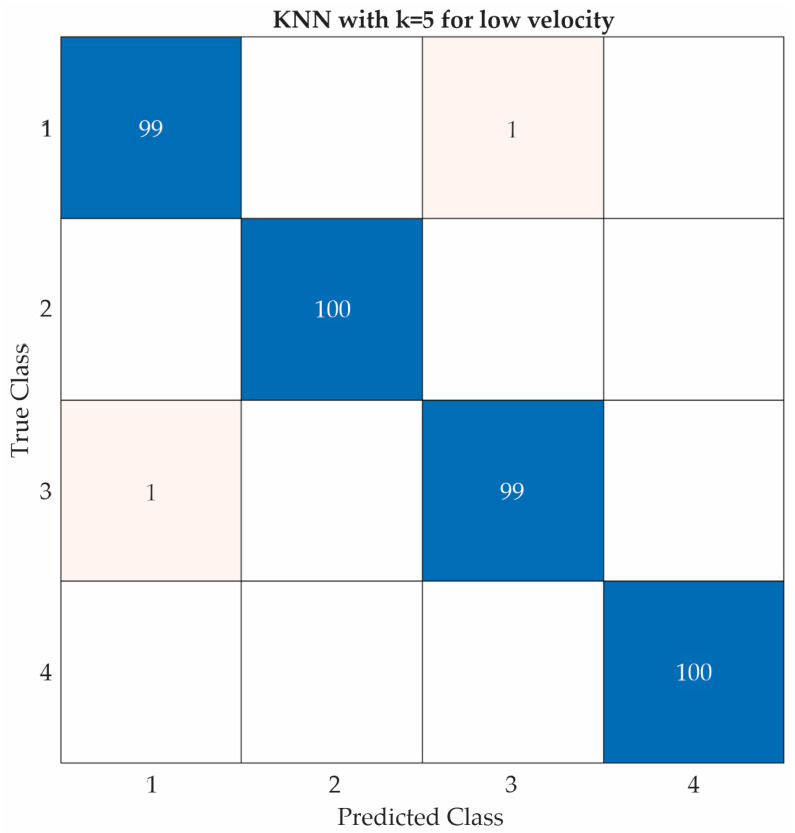
KNN confusion matrix for low velocity.

**Figure 18:**
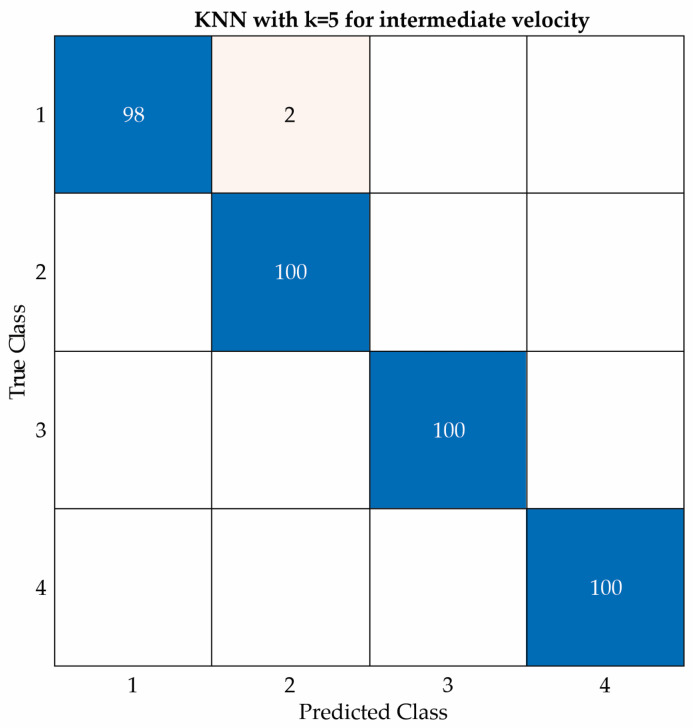
KNN confusion matrix for intermediate velocity.

**Figure 19:**
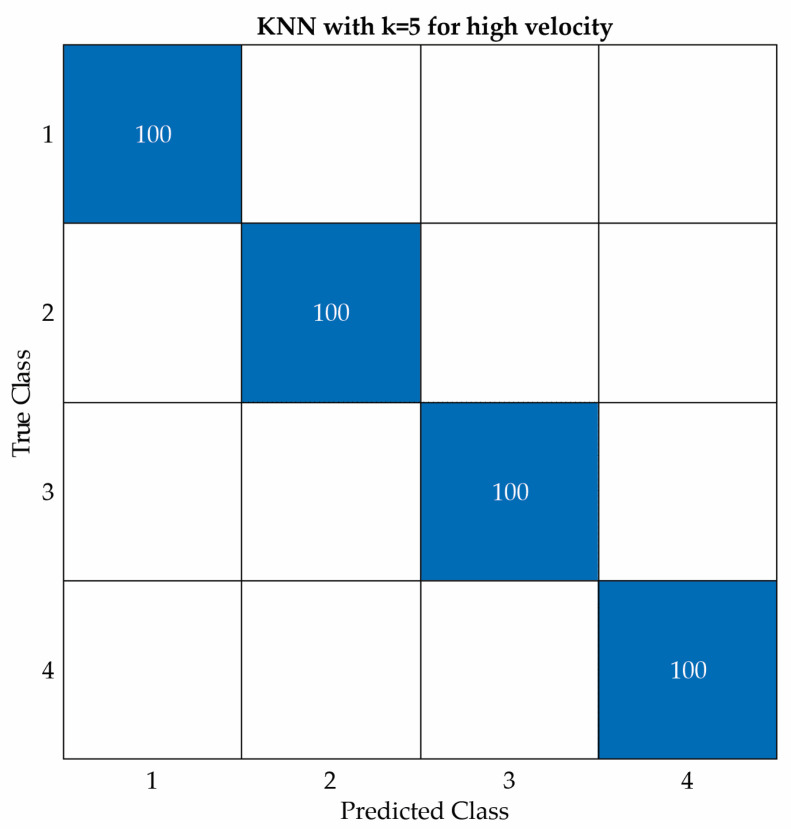
KNN confusion matrix for high velocity.

Figure 17, Figure 18 and Figure 19 show the confusion matrices of the KNN classifier for low, intermediate, and high velocities, respectively. This classifier obtained two errors at both a low velocity and an intermediate velocity, but zero errors at a high velocity.

Table 3 shows the classifier settings and results for all of the velocities. On the other hand, Table 4 shows the selected indicators for all the classifiers: the mean (orange color), RMS (green color), and Std (blue color) for the Y-axis. These indicators are determined by looking for similarities between the 10 best indicators of each velocity. This ensures that the same classifier and the same three indicators are the potential solution for diagnosing the severity of cracks in blades regardless of the velocity.

With these final results, it is important to mention that the computational time of the proposal is 0.077178 s considering the computation of the three SIs and the application of the KNN algorithm by using the hardware and software mentioned in Section 4.1. This value is not enough to operate in real time if the sampling frequency of 10,000 samples/s (or a sampling time of 0.0001 s) is considered. However, the proposal can be implemented into a parallel computing platform (e.g., using parallel platforms on MATLAB or FPGA technologies) to operate with its inherent batch processing time, i.e., the proposal is based on the batch processing of 1200 samples (or a time window of 0.12 s) of the vibration signal. Thus, the next vibration data window can be acquired and stored while the result of the previous data window is analyzed. In addition, operating in real time and as an integrated hardware solution (e.g., using a single sensor and/or a single hardware unit) is fundamental to applying opportune actions that minimize the negative impact of damages in WTs or any structure and contributing to the solution of current infrastructure monitoring demands worldwide [29].

## 5. Conclusions

Condition monitoring and early fault detection are of paramount importance in maintenance tasks for any system or piece of equipment. In WTs, the detection of cracks in blades can reduce repair costs and avoid more severe damages. In this work, a ML method to detect cracks in WT blades by using vibration signals is presented. The proposal is validated experimentally in a low-power WT by considering three levels of severity and three velocities. Although different severity levels with a higher resolution, e.g., mm by mm, were not tested, it was found that the proposed method can diagnose different severity levels. In a future work, the sensitivity of this method will be explored. Moreover, the three velocities analyzed in this work cover the entire operating range of the WT; therefore, it can be inferred that the proposed method can be adapted to any operating velocity of the WT.

Using the proposed ML method after the ANOVA test, it was found that the statistical indicators can provide important information to differentiate between various blade conditions, with the mean, RMS, and standard deviation of the Y-axis being the ones that provide the best results regardless of the velocity. With these indicators, four classifiers, i.e., DTs, NNs, SVMs, and KNNs, were tested, with the KNNs with k=5 being the one that provided the most accurate results, obtaining a 99.5% accuracy at low wind velocities and a 100% accuracy at high wind velocities. Although promising results were obtained through the proposed Matlab software tool, these have to be considered as preliminary since further research is needed.

In a future work, the proposed method will be applied and updated for time-varying wind profiles. Furthermore, other damages in a single or combined way will be investigated, mainly considering early or incipient damages. In addition, different simulations using the finite element method (FEM) will be also carried out in order to validate and strengthen the obtained results, as the proposed research work is based on experimentations in a controlled laboratory. Finally, the developed method will be implemented in FPGA hardware with the aim of providing a technological tool for online and real-time monitoring.

## Figures and Tables

**Table 1:** Dataset for the blade conditions.

Condition	Velocity	Axis X	Axis Y	Axis Z
Healthy	Low	100	100	100
	Intermediate	100	100	100
	High	100	100	100
Light damage	Low	100	100	100
	Intermediate	100	100	100
	High	100	100	100
Intermediate damage	Low	100	100	100
	Intermediate	100	100	100
	High	100	100	100
Severe damage	Low	100	100	100
	Intermediate	100	100	100
	High	100	100	100
	**Total**	**1200**	**1200**	**1200**

**Table 2:** One-way ANOVA results.

Feature	Axis	One-Way ANOVA
Mean	Y	45.898
Skewness	Y	28.7428
RMS	Y	26.7202
Std	Y	24.4612
Mean	Z	22.4664
SINAD	Y	19.7261
EMS	Z	18.9073
Shape factor	Z	16.2734
SNR	Z	15.4659
Std	Z	15.0407

**Table 3:** KNN settings and results.

	Low Velocity	Intermediate Velocity	High Velocity
**K value**	5	5	5
**Accuracy**	99.5%	99.5%	100%
**Prediction speed**	8500 observation/s	12,000 observation/s	8400 observation/s
**Training time**	18.253 s	18.785 s	21.945 s
**Distance metric**	Euclidean	Euclidean	Euclidean
**Distance weight**	Equal	Equal	Equal

**Table 4:** Features sorted for each velocity.

Low Velocity		Intermediate Velocity		High Velocity	
Feature	One-Way ANOVA	Feature	One-Way ANOVA	Feature	One-Way ANOVA
AxisY/Mean	45.898	AxisZ/Mean	18.0144	AxisY/RMS	1.41 × 10^3^
AxisY/Skewness	28.7428	AxisZ/RMS	13.3412	AxisY/Std	563.3356
AxisY/RMS	26.7202	AxisY/Mean	5.2677	AxisY/Mean	329.2885
AxisY/Std	24.4612	AxisY/Skewness	3.1481	AxisY/ShapeFactor	226.6165
AxisZ/Mean	22.4664	AxisY/PeakValue	2.4873	AxisY/PeakValue	214.3894
AxisY/SINAD	19.7261	AxisZ/Std	2.2055	AxisX/SNR	58.6886
AxisZ/RMS	18.9073	AxisZ/ShapeFactor	2.1075	AxisX/ShapeFactor	57.6459
AxisZ/ShapeFactor	16.2734	AxisY/Kurtosis	2.054	AxisY/ClearanceFactor	51.9998
AxisZ/SNR	15.4659	AxisY/RMS	1.9922	AxisZ/RMS	50.3171
AxisZ/Std	15.0407	AxisY/Std	1.9866	AxisX/Std	47.4861

## Data Availability

The data presented in this study are not publicly available.

## Abbreviations

The following abbreviations are used in this manuscript:

ANOVA	Analysis of Variance
DT	Decision Tree
EEMD	Ensemble Empirical Mode Decomposition
FFT	Fast Fourier Transform
FPGA	Field programmable gate array
KNN	K-Nearest Neighbor
ML	Machine learning
NN	Neural Network
RMS	Root Mean Square
RSSA	Recursive singular spectrum analysis
SINAD	Signal-to-Noise and Distortion ratio
SNR	Signal-to-noise Ratio
SVM	Support Vector Machine
THD	Total Harmonic Distortion
WT	Wind turbine
